# A novel polyomavirus from the nasal cavity of a giant panda (*Ailuropoda melanoleuca*)

**DOI:** 10.1186/s12985-017-0867-5

**Published:** 2017-10-27

**Authors:** Dunwu Qi, Tongling Shan, Zhijian Liu, Xutao Deng, Zhihe Zhang, Wenlei Bi, Jacob Robert Owens, Feifei Feng, Lisong Zheng, Feng Huang, Eric Delwart, Rong Hou, Wen Zhang

**Affiliations:** 1 Sichuan Key Laboratory of Conservation Biology for Endangered Wildlife, Chengdu Research Base of Giant Panda Breeding, Chengdu, Sichuan 610081 China; 2 Department of Microbiology, School of Medicine, Jiangsu University, Zhenjiang, Jiangsu 212013 China; 3 Shanghai Veterinary Research Institute, Chinese Academy of Agricultural Sciences, Shanghai, 200241 China; 4 Blood Systems Research Institute, San Francisco, California, 94118 USA; 5 Liziping Nature Reserve, YaAn, Sichuan Province, Sichuan, 625499 China

**Keywords:** Giant panda, Polyomavirus, Complete genome

## Abstract

**Background:**

Polyomaviruses infect a wide variety of mammalian and avian hosts with a broad spectrum of outcomes including asymptomatic infection, acute systemic disease, and tumor induction.

**Methods:**

Viral metagenomics and general PCR methods were used to detected viral nucleic acid in the samples from a diseased and healthy giant pandas.

**Results:**

A novel polyomavirus, the giant panda polyomavirus 1 (GPPyV1) from the nasal cavity of a dead giant panda (*Ailuropoda melanoleuca*) was characterized. The GPPyV1 genome is 5144 bp in size and reveals five putative open-reading frames coding for the classic small and large T antigens in the early region, and the VP1, VP2 and VP3 capsid proteins in the late region. Phylogenetic analyses of the large T antigen of the GPPyV1 indicated GPPyV1 belonged to a putative new species within genus *Deltapolyomavirus*, clustering with four human polyomavirus species. The GPPyV1 VP1 and VP2 clustered with genus *Alphapolyomavirus*. Our epidemiologic study indicated that this novel polyomavirus was also detected in nasal swabs and fecal samples collected from captive healthy giant pandas.

**Conclusion:**

A novel polyomavirus was detected in giant pandas and its complete genome was characterized, which may cause latency infection in giant pandas.

## Background

Members of the family *Polyomaviridae* are small viruses characterized by a non-enveloped icosahedral capsid and a circular double-stranded DNA genome of approximately 5000 bp. Early and late genes are transcribed bi-directionally, starting from a short non-coding regulatory region. Early genes encode two or three proteins, designated tumor antigens, which participate in viral genome replication and cellular transformation. The late genes encode the major and minor capsid proteins VP1, VP2, and VP3 [1]. In addition, some of the primate and human polyomaviruses encode an additional non-structural multifunctional protein, the agnoprotein [2]. The International Committee on Taxonomy of Viruses (ICTV) officially lists 73 species as polyomaviruses and divides the family into 4 genera including *Alpha*-, *Beta-*, *Gamma*- and *Deltapolyomavirus* [3].

Many mammalian polyomaviruses cause subclinical infections with life-long persistence in their immune competent hosts [4]. Only some of the polyomaviruses have been described to cause disease in immune-compromised hosts [5]. Polyomavirus-related diseases, which include nephritis [6], encephalitis [7], Merkel cell carcinoma [8], skin dysplasia [9, 10], and pneumonitis [11, 12], can occur among immune-suppressed individuals, including post-transplantation and AIDS patients. Polyomaviruses are widely distributed among mammalian and avian species and besides humans they have been identified in monkeys, cattle, rabbits, raccoons, rodents, bats, elephants, badgers, and a wide variety of birds [13–24].

In the present study, using viral metagenomics we detected a novel polyomavirus, the giant panda polyomavirus 1 (GPPyV1), in the nasal cavity of a giant panda (*Ailuropoda melanoleuca*) who died for unknown reasons after released into the wild.

## Materials and methods

### Samples

In July 2016, a two-year old captive giant panda was released to the wild environment of the Liziping Nature Reserve in YaAn, Sichuan Province, China. The giant panda was found dead on 27 September of the same year, having lost about 30% of its weight. Autopsy indicated that the gastrointestinal tract was complete empty and no feces were found near its body. The nasal secretion and tissues including heart, liver, spleen, lung, pancreas, muscle, testis, thyroid, and kidney tissues were collected for viral nucleic acid detection. All samples were collected by disposable materials and shipped on dry ice. The rayon ball of the nasal swab was put into the conical tube containing 1 mL PBS. The tube was then vigorously vortexed for 5 min and incubated for 30 min in 4 °C. The supernatants were then collected after centrifugation (10 min, 15,000×*g*). Tissue samples (~25 mg) were homogenized, frozen and thawed three times on dry ice, the supernatants were then collected after centrifugation (10 min, 15,000×*g*).

### Viral metagenomic analysis

500 μl of each supernatant was filtered through a 0.45-μm filter (Millipore) to remove eukaryotic and bacterial cell-sized particles. The filtrates enriched in viral particles were treated with DNase and RNase to digest unprotected nucleic acid at 37 °C for 60 min [25–27]. Remaining total nucleic acid was then isolated using QiaAmp Mini Viral RNA kit (Qiagen) according to manufacturer’s protocol. Four libraries were then constructed using Nextera XT DNA Sample Preparation Kit (Illumina) and sequenced using the MiSeq Illumina platform with 250 bases paired ends with dual barcoding for each library. Three libraries were constructed using tissue samples, each including three types tissues. One library consisted of the nasal swab sample. For bioinformatics analysis, paired-end reads of 250 bp generated by MiSeq were debarcoded using vendor software from Illumina. An in-house analysis pipeline running on a 32-nodes Linux cluster was used to process the data. Clonal reads were removed and low sequencing quality tails were trimmed using Phred quality score ten as the threshold. Adaptors were trimmed using the default parameters of VecScreen which is NCBI BLASTn with specialized parameters designed for adapter removal. The cleaned reads were de-novo assembled by SOAPdenovo2 version r240 using Kmer size 63 with default settings. The assembled contigs, along with singlets were aligned to an in-house viral proteome database using BLASTx with an E-value cutoff of <10^−5^ [28].

### Genome sequencing and PCR screening

After identifying polyomavirus sequences in the nasal swab, inverse PCR was used to generate the complete genome of the novel polyomavirus. The inverse primers were designed based on a 712 bases contig: InPoFP (5′-*G* AATGGTGTGGGCCCACTATG-3′) and InPoRP (5′-*G* TTTGTCCGCCAGTGTAGCTT-3′) were used for the 1st round PCR and InPoFF (5′- GATGCAGTACCGTGGATTGC-3′) and InPoRF(5′- TTGGAGGGATCAGGACACCA-3′) for the 2nd round PCR. Here, the bases with asterisk means phosphorothioation. In order to investigate the prevalence of the novel polyomavirus in the captive giant pandas, 13 nasal swabs and 25 fecal samples were collected from healthy captive giant pandas in Chengdu Panda breeding center, and were subjected to nested PCR screening using primers, also designed based on the 712 bp partial VP1 gene sequence, including PoscrFP (5′ TGGTGTCCTGATCCCTCCAA-3′) and PoscrRP (5′- TCCAGGCAAAGGCTCAGTTC-3′) for the 1st round PCR and IPoscrFF (5′- AAGCTACACTGGCGGACAAA-3′) and PoscrRF(5′- CTGACCTTCCATTGTGGGCA-3′) for the 2nd round PCR. The original polyomavirus positive nasal swab sample was used as positive control in the PCR screening. Sanger method was used for sequencing of the inverse and regular PCR products.

### Phylogenetic analysis

Phylogenetic analyses were performed based on the predicted amino acid sequence in the present study, their closest viral relatives based on the best BLASTp hits in GenBank, and representative members of related viral species or genera. Sequence alignment was performed using CLUSTAL W with the default settings. Phylogenetic trees with 500 bootstrap resamples of the alignment data sets were generated using the Maximum-likelihood (ML) method in MEGA5.0. Putative ORFs in the viral genome were predicted by combining Geneious 8.1 software and NCBI ORF finder. Putative exon and intron were predicted by Netgenes2 at http://www.cbs.dtu.dk/services/NetGene2/.

### Nucleotide sequence accession numbers

The novel polyomavirus genome of GPPyV1 and sequence generated by PCR screening were deposited in GenBank under the following accession numbers: KY612371 and MF370864.

## Results

DNA libraries from samples of giant pandas were generated and sequenced using the lllumina MiSeq platform. In the nasal sample 17 sequence reads generating 3 different contigs were found to have strong amino acid homology to polyomaviruses. PCR screening of all the samples from the dead wild giant panda, plus 30 other nasal swab samples and 200 fecal samples collected from healthy captive giant pandas in the the Chengdu Research Base of Giant Panda Breeding in China with a set of nested primers designed on the VP1 sequence. Results indicated that one nasal swab and two fecal samples from healthy captive giant pandas and only the nasal swab from the dead animal were positive, which suggests that polyomavirus may establish latency infection in the gastrointestinal tract and nasal cavity of giant pandas.

**Fig. 1:**
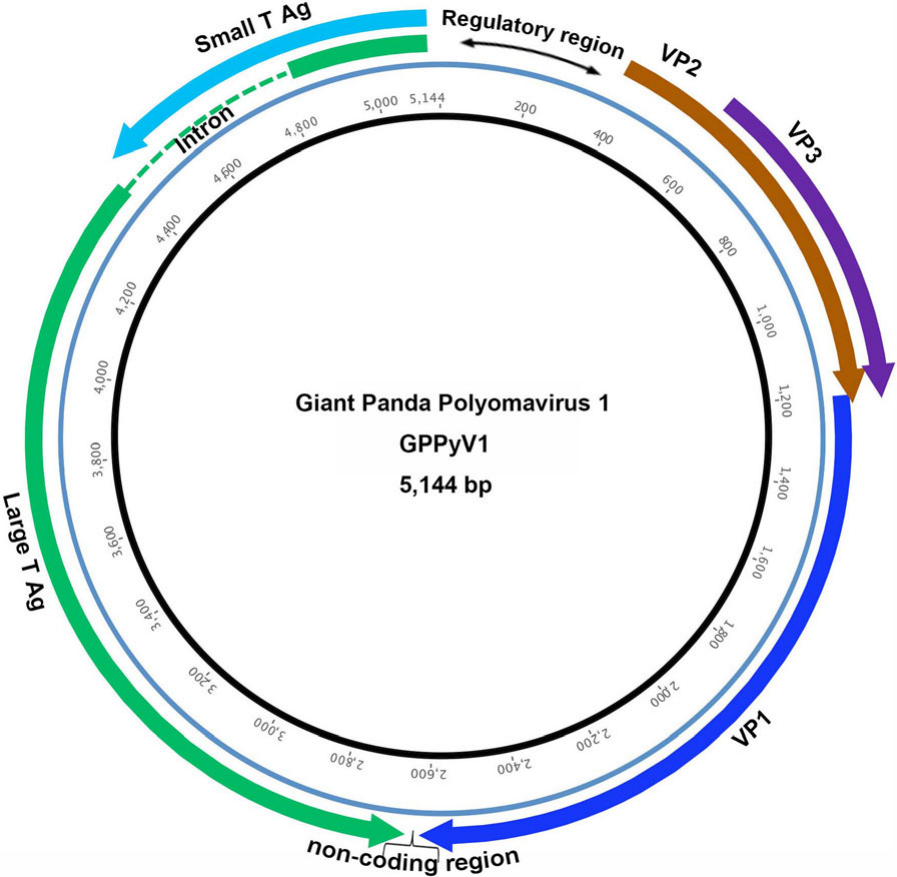
Schematic of the giant panda polyomavirus 1 (GPPyV1) genome organization

Sequence analysis based on the 299 bp fragments generated PCR screening, the 3 sequences from the healthy giant pandas were identical and showed only one nucleotide difference from the sequence from the died giant panda, suggesting these polyomavirus sequences may be from the same ancestor virus which was prevalent in the giant panda group. Using inverse PCR, the complete genome was then acquired and sequenced by Sanger walking from the nasal sample of the dead giant panda. The circular genome of the giant panda polyomavirus (named GPPyV1) was 5144 bases, with the overall GC content of 41.8%, which is similar to those of BK (39%), JC (40%), WU (39%) and other polyomaviruses [29]. The genome organization includes an early region coding on one strand for the small T antigen (STAg) and the large T antigen (LTAg), and a late region coding on the opposite strand for the capsid proteins VP1, VP2, and VP3, with a noncoding regulatory region between the beginning of the early region and the beginning of the late region, homologous to earlier described polyomaviruses (Fig. 1). Two copies of the consensus pentanucleotide LTAg binding site GAGGC, or the reverse complement GCCTG were present in the a noncoding regulatory region, where the sequence is GAGGCAGAAGGCCTC which might represent the possible core of the origin of replication [2]. The noncoding region between the ends of the late and early regions (Fig. 1) showed an AT-rich region. The sizes of the proteins deduced from the genome sequences of GPPyV1, their calculated molecular weights and isoelectric points are shown in Table 1.

**Table 1:** Size and position of predicted open reading frames (ORF) of GPPyV1, the predicted molecular masses (kiloDalton), and the isoelectric point (pI) of the translated proteins

ORF	Position	Length		MW (kDa)	pI
		nt	aa		
URR*	1–354	n/a	n/a	n/a	n/a
VP2	355–1278	924	307	33.69	4.73
VP3	571–1278	708	235	26.55	6.37
VP1	1247–2575	1329	442	48.07	6.88
NCR**	2576–2616	n/a	n/a	n/a	n/a
LTAg	2617–4548	2172	723	81.57	6.38
	4905–5144				
STAg	4566–5144	579	192	22.54	8.43

^*^Upstream regulatory region

^**^Noncoding region

A more detailed analysis of the amino acid sequences of GPPyV1 revealed conserved sequences in functionally important regions of the encoded proteins. The proteins encoded by the 5 GPPyV1 open-reading frames contain the typical elements that are necessary to accomplish their function in the viral life cycle of polyomaviruses. The unspliced early mRNA of GPPyV1 encodes the STAg, which is 192 aa in length, where the CXCX_2_C consensus sequence for protein phosphatase 2A binding is present. The initial 80 amino acids of the N-terminus of the STAg and LTAg of GPPyV1 are identical, which is a general characteristic in all polyomaviruses. The LTAg is generated by partial splicing of the early mRNA transcript. Conserved features like the J-domain, which is important for efficient DNA replication and transformation, is found in the LTAg sequence. This domain contains the highly conserved HPDKGG box and a pRB-binding motif LXCXE, which are crucial for DNA replication [9].

**Fig. 2:**
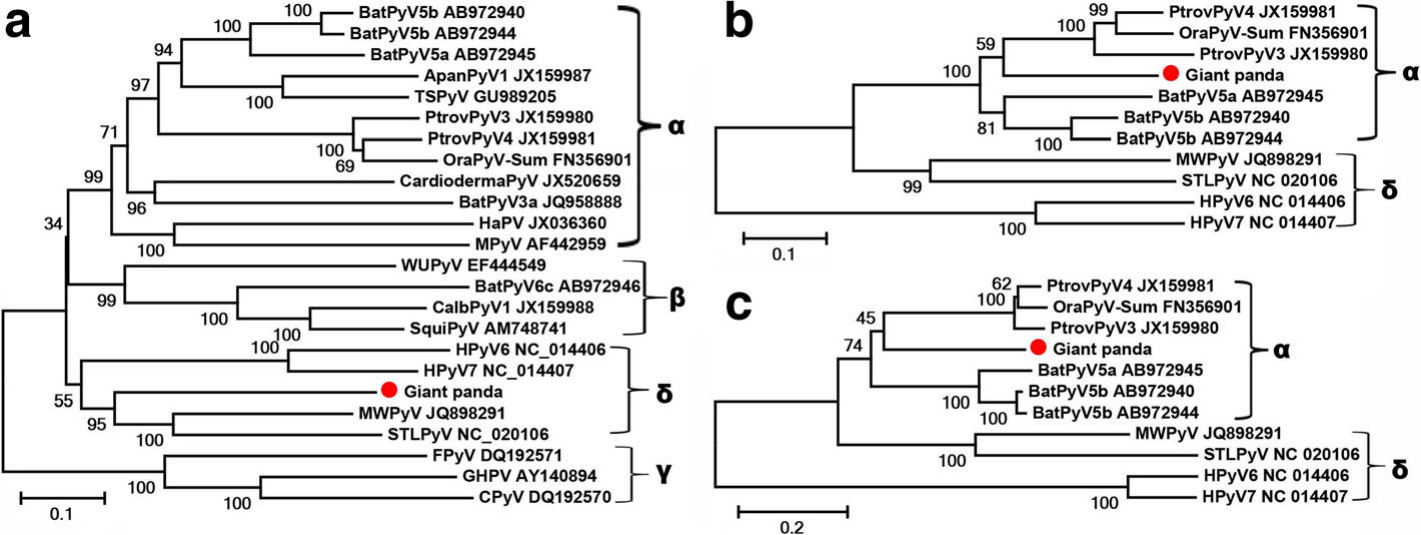
Amino acid-based maximum likelihood analysis of (**a**) the large T antigen, (**b**) the major capsid protein VP1, and (**c**) the minor capsid protein VP2. The polyomavirus identified in this study was labeled with a red dot

To determine the genetic relationship between GPPyV1 and the other related polyomaviruses. Phylogenetic analysis based on the putative LTAg amino acid sequences of polyomaviruses, including GPPyV1, the representative polyomavirus species of the four genera, and the best BLASTp searching matches of GPPyV1 in GenBank, were performed and maximum-likelihood (ML) tree was generated. Results indicated that GPPyV1 is highly divergent from these other polyomaviruses and that it fell within the clade of genus *Deltapolyomavirus* together with four species from human, sharing the highest amino acid sequence identity of 53% of with MW polyomavirus (GenBank no. JQ898291) (Fig. 2A). BLASTp searches in GenBank based on the amino acid sequences of VP1 and VP2 indicated GPPyV1 showed the closest relationship with polyomaviruses within genus *Alphapolyomavirus* which includes bat polyomavirus 5 (BatPyV-5) and *Pan troglodytes* verus polyomavirus 3 and 4 (PtroPyV3 and PtroPyV4), sharing 67–68% and 56–57% with them, respectively. ML trees were then established based on the amino acid sequence of VP1 and VP2, respectively, from the representative strains in genus *Deltapolyomaviru*s and genus *Alphapolyomavirus.* Both trees revealed that GPPyV1 clustered within the clade of genus *Alphapolyomavirus,* discordant with the tree based on LTAg (Fig. 2 B and C), suggesting this ployomavirus strain from giant panda was involved in genomic recombination which frequently occurred during polyomavirus evolution [30].

## Discussion

Recently, the ICTV Polyomaviridae Study Group designed the new criteria for definition of a new polyomavirus species [3], which includes four major points: i) the complete genome sequence is available in public databases and published; ii) the genome displays an organization typical for polyomaviruses; iii) sufficient information on the natural host is available; iv) the observed genetic distance to members of the most closely related species is >15% for the large T antigen (LTAg) coding sequence. Based on these criteria, GPPyV1 qualifies as a novel polyomavirus species.

In the present study, using viral metagenomic method, a new polyomavirus was detected in the nasal cavity of a dead giant panda recently released into the wild who died from unknown causes. Our PCR analysis of 13 nasal swabs and 25 fecal samples from healthy captive giant pandas showed no other positive animals, suggesting GPPyV1 is not highly prevalent in captive giant pandas. In mammals polyomaviruses infections are highly common during childhood and adolescence [31]. These persistent infections are not generally associated with acute disease in their natural non-immunocompromised hosts. On the other hand, mammalian polyomaviruses are known to induce tumors after inoculation into non-permissive laboratory rodents [32] and recently, the human polyomavirus, Merkel cell polyomavirus, has been linked to the development of skin carcinoma [8, 33]. In this study, phylogenetic analysis based on LTAg of GPPyV1 showed it had the closest relationship with polyomaviruses from genus *Deltapolyomaviru*s which currently includes four defined species: HPyV-6, HPyV-7, MW polyomavirus, and STL polyomavirus. HPyV-6 and HPyV-7 were mainly found on human skin, while MW polyomavirus and STL polyomavirus have been isolated out of human feces, some of which are associated with human disease, for example, HPyV-7 was associated pruritic rash and viremia in transplant recipients, while MW and STL polyomavirus were reported to be present in tonsillar tissues from children with chronic tonsillar disease [10, 34]. Using an alternative classification scheme both the LT and the VP1 proteins cluster in the Almi clade [35]. The polyomaviruses (HPyV-6, HPyV-7, MW, and STL) whose LT are most closely related to that of GPPyV1 have VP1s that cluster in a different (Wuki) clade than GPPyV1 (Almi) indicating that these genomes may be recombinant of an original virus whose VP1 gene was more related to the GPPyV1 genome described here. During the evolution of polyomavirus, recombination does not seem to occur randomly across the genome but has rearranged the early and late genomic regions of major polyomavirus lineages. For instance, a lineage comprising KIPyV, WUPyV and two related rodent polyomaviruses belongs to the clade corresponding to the genus Betapolyomavirus in LTAg phylogenetic trees, however it clusters within a lineage of the genus Deltapolyomavirus based on the phylogenetic analysis over the VP1 sequences [35–37].

## Conclusion

A novel polyomavirus, GPPyV1, was detected in the nasal cavity of a dead giant panda, whose complete genome was determined. Phylogenetic analysis based on the LTAg suggested GPPyV1 belonged to a putative new species within genus *Deltapolyomaviru*s. Epidemiological results suggested GPPyV1 is also prevalent in captive giant pandas. Whether GPPyV1 cause specific disease in giant pandas remain unknown.

## Abbreviations

BLAST: Basic Local Alignment Search Tool
GPPyV: giant panda polyomavirus
ICTV: the International Committee on Taxonomy of Viruses
LTAg: large T antigen
ORF: open reading frame
PCR: polymerase chain reaction
STAg: small T antigen
